# In Vivo Assessment of Natural Killer Cell Responses during Chronic Feline Immunodeficiency Virus Infection

**DOI:** 10.1371/journal.pone.0037606

**Published:** 2012-05-31

**Authors:** Rita D. Simões, Kristina E. Howard, Gregg A. Dean

**Affiliations:** Center for Comparative Medicine and Translational Research, College of Veterinary Medicine, North Carolina State University, Raleigh, North Carolina, United States of America; Auburn University, United States of America

## Abstract

Accumulating evidence suggests that natural killer (NK) cells may have an important role in HIV-1 disease pathogenesis; however, in vivo studies are lacking. Feline immunodeficiency virus (FIV) infection of cats provides a valuable model to study NK cell function in vivo. The immune response against *Listeria monocytogenes* (Lm) is well characterized, allowing its use as an innate immune probe. We have previously shown that locally delivered IL-15 can improve Lm clearance in FIV-infected animals, and this correlated with an increase in NK cell number. In the present study, chronically FIV-infected and SPF-control cats were challenged with Lm by unilateral subcutaneous injection next to the footpad and then treated with 5-bromo-2′-deoxyuridine (BrdU). The Lm draining and contralateral control lymph nodes were evaluated for NK, NKT, CD4^+^ and CD8^+^ T cell number, proliferation, apoptosis, and NK cell function. *Listeria monocytogenes* burden was also assessed in both control and Lm draining lymph nodes. NK, NKT, CD4^+^ T and CD8^+^ T cells in the Lm-challenged lymph node of FIV-infected cats did not increase in number. In addition, after Lm challenge, NK cells from FIV-infected cats did not increase their proliferation rate, apoptosis was elevated, and perforin expression was not upregulated when compared to SPF-control cats. The failure of the NK cell response against Lm challenge in the draining lymph node of FIV-infected cats correlates with the delayed control and clearance of this opportunistic bacterial pathogen.

## Introduction

Natural killer (NK) cells are part of the innate immune compartment and are considered the first line of defense against obligate intracellular pathogens and transformed cells. Recent studies have shown the importance of NK cells as a bridge between innate and adaptive immune responses, and that in collaboration with other innate immune cells they help modulate the type and strength of the adaptive immune response (reviewed in [1]). Several studies have suggested the NK cell response during the course of HIV-1 infection is compromised. Significant abnormalities in NK cell phenotype, function and number have been reported during HIV-1 infection [2], [3]. Mechanisms have been proposed to explain the NK cell defect in HIV-1 infection, including reduction of T cell-derived IL-2, induction of apoptosis, and modulation of MHC class I receptors by NK cells [4], [5]. Furthermore, the importance of NK cells in HIV-1 infection has been corroborated by studies showing that certain combinations of killer immunoglobulin-like receptors (KIR) and MHC class I molecules correlate with a slower HIV-1 disease progression [6], while HIV-1 exposed healthy subjects show enhanced NK cell function [7]. Although there is convincing evidence supporting the importance of NK cells during the course of HIV-1 infection, the exact mechanisms underlying NK cell dysfunction are unknown. Since investigating the dynamics of the NK cell response in lymph nodes (LN) of HIV-infected or healthy people in response to a microorganism challenge is not feasible, we used the feline immunodeficiency virus (FIV) model to study HIV/AIDS. FIV infection of cats is clinically and immunologically similar to HIV-1 in people [8]–[10], providing a valuable animal model to investigate the consequences of lentivirus infection on the innate immune response. Because the innate immune response to *Listeria monocytogenes* (Lm) is well understood (reviewed in [11]), we used this intracellular pathogen to probe the innate immune system in order to investigate the effects of chronic FIV infection on NK cell function. We previously reported that FIV-infected cats have an impaired innate response that fails to gain initial control of bacterial replication prior to the adaptive immune response [12]. We also demonstrated that locally delivered IL-15, a cytokine known to activate and stimulate NK cell proliferation, cytolytic activity, and cytokine and chemokine production, significantly restored innate immune function as measured by Lm clearance [13]. Here, we show that compared to SPF-control cats, NK cells from chronically FIV-infected cats have a constitutively higher level of proliferation that is counter-balanced by increased apoptosis. Upon challenge with Lm, NK cells of FIV-infected cats fail to traffic to lymph nodes, have a lower proliferative response, and show a minimal increase in perforin expression.

## Results

### Innate Immune Control of Lm is Impaired in Chronically FIV-infected Cats

We have previously shown that chronically and acutely FIV-infected cats have an impaired innate immune response to the intracellular pathogen Lm [12],[13]. Here we showed that 3 days post-Lm challenge, chronically FIV-infected animals had a greater number of Lm colony-forming units per LN than SPF-control cats (64,280±31,253; 5,318±3,878 CFU/LN respectively, mean ± SEM). No bacterial colonies were recovered from the contralateral control LN regardless of FIV status (data not shown). Plasma viremia from chronic FIV-infected cats ranged from 471 to 5121 copies/mL, and FIV proviral load ranged between 245 and 7345 per 1×10^6^ PBMC (data not shown). These results confirmed that the innate immune response against Lm in cats with chronic FIV infection is diminished compared to SPF-control animals.

#### Chronic FIV infection is associated with decreased NK, NKT, CD4^+^ T and CD8^+^ T cell numbers in Lm challenged LN

**Figure 1:**
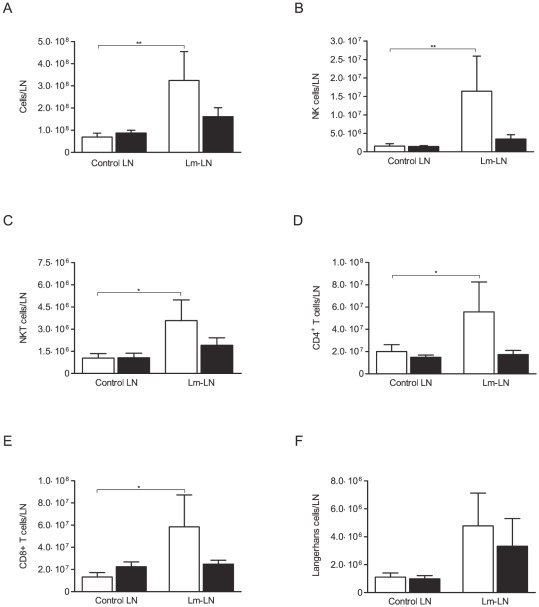
Chronic FIV infection is associated with decreased lymphocyte numbers in Lm challenged LN. Chronically FIV-infected and SPF-control cats were challenged with 2.5×10^5^ cfu *Listeria monocytogenes* subcutaneously proximal to either the right metatarsal or metacarpal footpad. After 3 days, the local draining lymph node and the contralateral control nodes were removed, processed into a single cell suspension, assessed by trypan blue dye exclusion, and the total number of cells per lymph node was determined. The absolute cell number for lymphocyte subpopulations was calculated based on the percent of gated lymphocytes determined by flow cytometric analysis and total number of lymphocytes from the LN. (A) Total cell number, (B) Absolute number of NK cells (CD3^−^CD56^+^), (C) Absolute number of NKT cells (CD3^+^CD56^+^), (D) Absolute number of CD4^+^ T cells (CD3^+^CD4^+^), (E) Absolute number of CD8^+^ T cells (CD3^+^CD8^+^), and (F) Absolute number of Langerhans cells (CD1a^+^). Columns represent mean ± standard error of the mean (SEM). FIV-infected (black columns) and SPF-control animals (white columns). Statistical significance was determined between control and Lm challenged LN. Statistical analysis was performed using Wilcoxon Signed-Rank test. * indicates *P*<0.05, ** indicates *P*<0.01. SPF-control cats n = 8, FIV-infected cats n = 13.

We previously reported that FIV-infected cats had a delayed and blunted enlargement and follicle formation in draining LN after Lm challenge as compared to SPF-control cats [12], [13]. To further investigate this, LN were removed on day 3 after Lm challenge and the total number of cells was determined. Lm-challenged LN from SPF-control cats contained a significantly greater number of cells as compared to control LN (*p*<0.01), but no such difference was observed in chronic FIV-infected cats (Figure 1A). These results show a quantitative difference in cellular response to Lm challenge by FIV-infected versus SPF-control cats.

**Figure 2:**
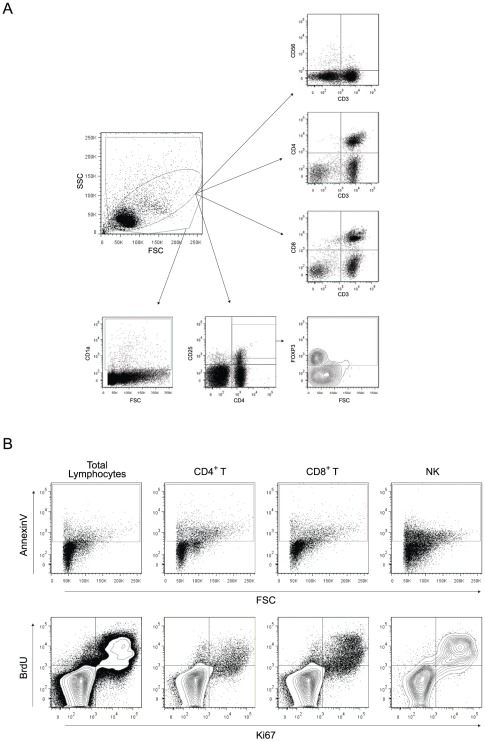
Flow cytometry gating strategy and representative plots. Flow cytometry gating strategy and representative plots from control lymph node cells of a SPF-control cat (CD4^+^ and CD8^+^ T cell and NK cell gatings) and from control lymph node of a FIV-infected cat (CD4^+^CD25^+^ and FOXP3 gatings). (A) The lymphocyte population was gated based on side and forward scatter (inner gate). Lymphocyte subpopulations were gated based on expression of CD3, CD4, CD8, CD56, CD25 and FOXP3. A broader gating strategy of the total cell population (outer gate) was used to identify the CD1a^+^ subpopulation. (B) Representative dot plots are shown for AnnexinV^+^ cells within total, CD4^+^ and CD8^+^ T cells, and NK cells. Representative contour plots of BrdU^+^ and Ki67^+^ cells within total, CD4^+^ and CD8^+^ T cells, and NK cells from a FIV-infected cat are shown.

To further characterize the reduced number of lymphocytes in the Lm draining lymph nodes of FIV-infected cats we determined the total cell number of NK cells (CD3^−^CD56^+^), NKT cells (CD3^+^CD56^+^), CD4^+^ T cells (CD3^+^CD4^+^), CD8^+^ T cells (CD3^+^CD8^+^), regulatory T cells (CD4^+^CD25^+^FOXP3^+^) and Langerhans cells (CD1a^+^) in LN of FIV-infected and SPF-control cats by flow cytometry. No differences in total NK cell numbers were observed between SPF-control and FIV-infected cats in control LN (Figure 1B). However, while SPF-control cats showed a 10-fold increase in NK cell number from Lm-challenged LN compared to control nodes, no such increase was observed between LN of FIV-infected animals (Figure 1B). This suggests FIV infection may affect NK cell recruitment, death and/or proliferation. SPF-control cats had a significant increase (*p*<0.05) in NKT, CD4^+^ and CD8^+^ T cell numbers in Lm-challenged nodes as compared to control LN (Figures 1C, D, E). Again, no such differences were seen between the nodes from FIV-infected cats. Interestingly, there were no differences in Langerhans cell number between challenged and control nodes or between FIV-infected and SPF-control cats (Figure 1F). We also found no difference in CD4^+^CD25^+^FOXP3^+^ cell number between LN, independent of the FIV status (data not shown). The percentages of the different cell populations observed within the control and Lm-challenged lymph nodes of both SPF-control and FIV-infected cats are shown in Figure S1A-E. While NK (Figure S1A) and CD8^+^ T cells (Figure S1D) cells showed a similar trend as seen with the absolute number of cell results, no differences were observed between the other cell populations analyzed. Flow cytometric gating strategies and representative plots are shown in Figures 2A and 2B, respectively.

**Figure 3:**
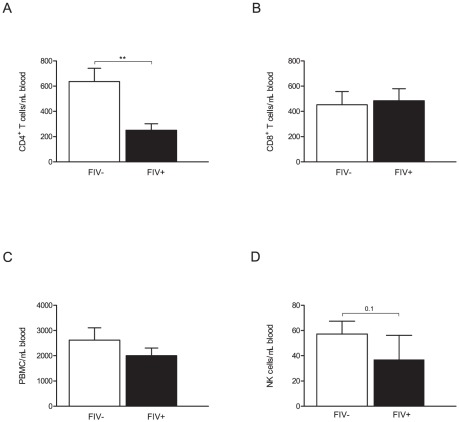
Effect of FIV infection on PBMC subpopulations. Whole blood was collected at the time of lymph node biopsy 3 days after the Lm challenge. The absolute cell number for lymphocyte subpopulations was calculated based on the percent of gated lymphocytes determined by flow cytometric analysis multiplied by lymphocyte absolute number. (A) Absolute number of CD4^+^ T cells, (B) Absolute number of CD8^+^ T cells, (C) Absolute number of PBMC, (D) Absolute number of NK cells. Columns represent mean ± SEM. FIV-infected (black columns) and SPF-control animals (white columns). Statistical significance was determined between control and Lm challenged LN. Statistical analysis was performed using Mann-Whitney U test. ** indicates *P*<0.01. SPF-control cats n = 8, FIV-infected cats n = 13.

Both FIV-infected and SPF-control cats were challenged subcutaneously with Lm and whole blood was collected at day 3 after challenge at the same time as LN collection. Circulating CD4^+^ T cell number was significantly lower in FIV-infected cats compared to SPF-control cats (Figure 3A). No differences were seen between circulating CD8^+^ T cell or total lymphocyte numbers (Figure 3B–C), whereas NK cell numbers in the blood compartment were lower in FIV-infected cats but did not reach significance (*p* = 0.1, Figure 3D). Percentagewise, SPF-control cats had twice as many circulating CD4^+^ T cells when compared to FIV-infected animals (Figure S2A) while FIV-infected cats had 1.5 more CD8^+^ T cells (Figure S2B) than SPF-control cats, and no differences were observed in circulating NK cells between both groups of cats (Figure S2C).

### NK Cell Proliferation Differs Depending on the Compartment Analyzed

Increased lymphocyte proliferation and turnover of T, B, and NK cells is characteristic of pathogenic lentivirus infection [14], [15]. We asked whether the proliferation rate of lymphocyte subpopulations in FIV-infected cats would increase in response to an immune challenge. Proliferation of NK cells, and CD4^+^ and CD8^+^ T cells, was measured by the in vivo incorporation of BrdU [16] as well as intranuclear Ki-67 expression [17].

**Figure 4:**
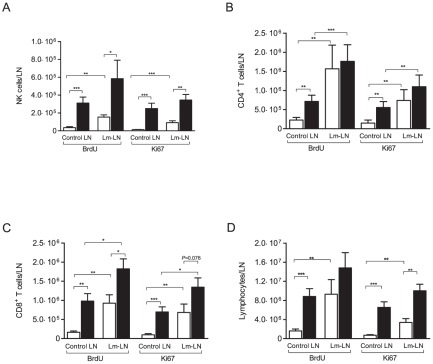
Effect of FIV infection on lymph node cell proliferation. Cell proliferation was assessed by BrdU incorporation and expression of the nuclear antigen Ki-67. The absolute number of proliferating lymphocyte subpopulations was calculated based on the percent of gated lymphocytes that either incorporated BrdU or expressed Ki-67, determined by flow cytometric analysis, and the total number of lymphocytes from the LN. (A) Absolute number of proliferating NK cells, (B) Absolute number of proliferating CD4^+^ T cells, (C) Absolute number of proliferating CD8^+^ T cells, (D) Absolute number of proliferating cells. Columns represent mean ± SEM. FIV-infected (black columns) and SPF-control animals (white columns). Statistical significance was determined between control and challenged LN, and between FIV-infected and SPF-control cats. Statistical analysis was performed using Wilcoxon Signed-Rank test and Mann-Whitney U test. * indicates *P*<0.05, ** indicates *P*<0.01, *** indicates *P*<0.001. SPF-control cats n = 8, FIV-infected cats n = 13.

Cell proliferation was assessed in control and challenged LN, as well as in PBMC. In FIV-infected cats, a higher number of NK cells incorporated BrdU and expressed Ki-67 in control and challenged LN as compared to LN from SPF-control cats (Figure 4A). However, SPF-control animals had a significantly greater increase in NK cell proliferation in response to Lm when compared to the contralateral control LN (*p*<0.01), while no significant difference in response to Lm was observed between the nodes of FIV-infected cats (Figure 4A). CD4^+^ T cell proliferation, as indicated by either BrdU incorporation or Ki-67 expression, was increased in the control but not the challenged LN of FIV-infected cats as compared to SPF-control cats (Figure 4B). In contrast to the NK cell response, CD4^+^ T cell proliferation in response to Lm was increased in both SPF-control and FIV-infected cats (Figure 4B). Similar results were observed with the CD8^+^ T cell population (Figure 4C). There was a significant difference in total lymphocyte proliferation between FIV-infected and SPF-control cats in control and challenged nodes (p<0.01, Figure 4D). Total lymphocyte proliferation was significantly increased (4 and 6 folds, Ki67 and BrdU, respectively) in challenged nodes of SPF-control cats compared to control nodes while no such difference was observed between lymph nodes of FIV-infected cat (Figure 4D). Similar results were observed regarding the percentage of proliferation for the different cell populations analyzed (Figure S3).

**Figure 5:**
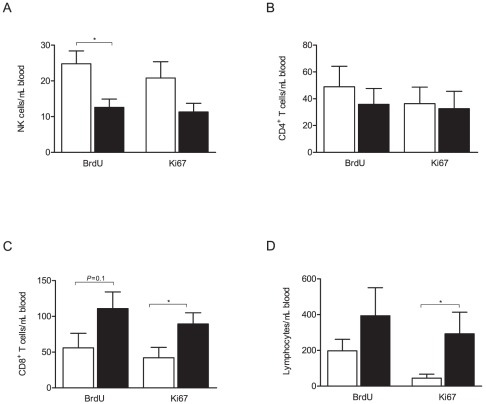
Effect of FIV infection on peripheral blood mononuclear cell (PBMC) proliferation. Whole blood was collected at the time of lymph node biopsy 3 days after the Lm challenge. Cell proliferation was assessed by BrdU incorporation and expression of the nuclear antigen Ki-67. (A) Absolute number of proliferating NK cells, (B) Absolute number of proliferating CD4^+^ T cells, (C) Absolute number of proliferating CD8^+^ T cells, (D) Absolute number of proliferating cells. Columns represent mean ± SEM. FIV-infected (black columns) and SPF-control animals (white columns). Statistical significance was determined between FIV-infected and SPF-control cats. Statistical analysis was performed using Mann-Whitney U test. * indicates *P*<0.05. SPF-control cats n = 8, FIV-infected cats n = 13.

Peripheral blood was collected 3 days after Lm challenge from both SPF-control and FIV-infected cats. In the blood compartment, 2 times more NK cells from SPF-control cats incorporated BrdU than in FIV-infected cats (Figure 5A). CD4^+^ T cell proliferation did not differ between FIV-infected and SPF-control animals (Figure 5B), while CD8^+^ T cell and total lymphocyte proliferation were significantly increased (2 and 6 fold, respectively) in FIV-infected cats (Figures 5C and 5D). Similar results were seen when the percentage of proliferation within the circulating cells was analyzed (Figure S4).

### Lymphocytes from FIV-infected Cats Undergo Apoptosis at a Higher Rate

**Figure 6:**
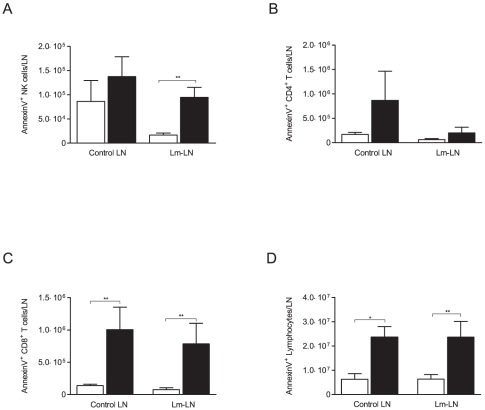
Lymphocyte subpopulations from FIV-infected cats undergo apoptosis at a higher rate. Apoptosis was assessed by AnnexinV^+^ staining followed by flow cytometric analysis. The absolute number of AnnexinV^+^ lymphocyte in each phenotypic subpopulations was calculated by determining the percent of each subpopulation by flow cytometric analysis and then multiplying by the total number of lymphocytes in the LN. (A) Absolute number of AnnexinV^+^ NK cells, (B) Absolute number of AnnexinV^+^ CD4^+^ T, (C) Absolute number of AnnexinV^+^ CD8^+^ T cells, (D) Absolute number of AnnexinV^+^ lymphocytes. Columns represent mean ± SEM. FIV-infected (black columns) and SPF-control animals (white columns). Statistical significance was determined between FIV-infected and SPF-control cats. Statistical analysis was performed using Mann-Whitney U test. * indicates *P*<0.05, ** indicates *P*<0.01. SPF-control cats n = 5, FIV-infected cats n = 6.

To reconcile the overall trend of lower lymphocyte numbers in the face of greater proliferation, we investigated whether cells were undergoing apoptosis as indicated by AnnexinV binding. FIV-infected cats had a higher number of apoptotic NK cells in the Lm-challenged LN as compared to SPF-control cats; however no difference was observed between the control nodes (Figure 6A). There was a trend that did not reach statistical significance of more CD4^+^ T cells undergoing apoptosis in control and challenged LN from FIV-infected cats (Figure 6B). CD8^+^ T cells apoptosis was greater in control and challenged LN (7 and 10 fold respectively) of FIV-infected as compared to SPF-control cats (Figure 6C). Similarly, the apoptotic rate of the total lymphocyte population was 3.5 fold higher in FIV-infected than in SPF-control cats in both control and Lm-challenged LN (Figure 6D). Percentagewise, a similar trend seen in absolute number of cells was observed (Figure S5A–C), with the exception of AnnexinV^+^ within the total lymphocyte population from CLN and Lm–LN (Figure S5D), where no differences were observed between SPF-control and FIV-infected animals. Interestingly, in the blood compartment, no differences in apoptosis were observed for any cell type investigated (data not shown).

### Fewer NK Cells from FIV-infected Cats Produce Perforin

**Figure 7:**
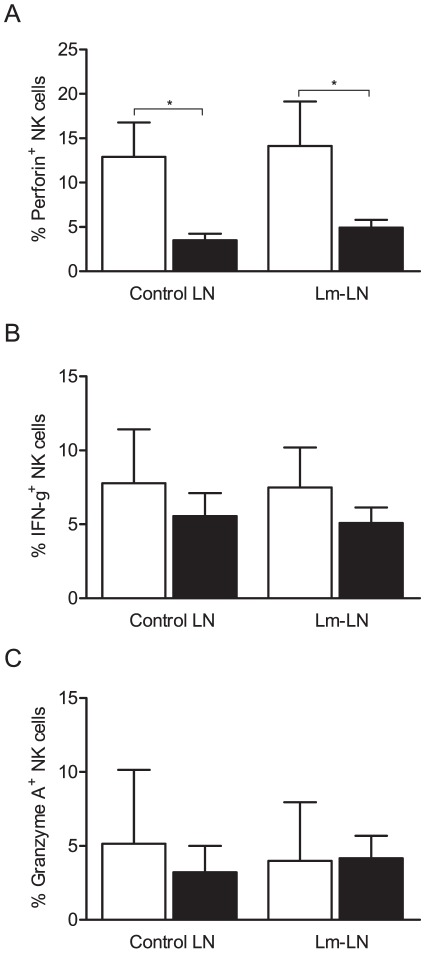
Fewer NK cells from FIV-infected cats produce perforin. Whole cell suspensions from lymph nodes were cultured overnight with IL-2. Cells were then co-cultured for 4 h with target cells at an effector:target ratio of 50∶1, with monensin added after the first hour of incubation. Cells were stained for cell surface receptors CD3 and CD56 and for intracellular perforin, IFN-γ and granzyme A. Samples were immediately analyzed by flow cytometry. Columns represent mean ± SEM. FIV-infected (black columns) and SPF-control (white columns). Statistical significance was determined between FIV-infected and SPF-control cats. Statistical analysis was performed using Mann-Whitney U test. * indicates *P*<0.05. SPF-control cats n = 6, FIV-infected cats n = 12.

As an indicator of NK cell functionality, we determined the number of NK cells expressing perforin, IFN-γ and granzyme A after ex vivo stimulation with IL-2. While a significantly higher number of Lm-challenged NK cells from SPF-control cats produced perforin after in vitro stimulation compared to FIV-infected cats (Figure 7A), we found no differences between FIV-infected and SPF-control cats with regard to expression of IFN-γ (Figure 7B) or granzyme A (Figure 7C).

## Discussion

The effect of HIV-1 infection on NK cells and the role of NK cells in HIV-1 control and immunopathogenesis have been the focus of increasing interest. Several findings point to a central role of NK cells in protection against HIV-1 and disease progression [6], [7], [18], and evidence suggests this cell population can be profoundly impaired during all the stages of HIV-1 infection in humans [2], [3], [19]. However, the mechanisms of NK cell dysfunction observed in HIV-1 patients are not fully understood and the impact on the overall immune response in vivo is unknown. In this study, using the FIV/cat model of HIV infection, we demonstrated that in vivo NK cell number, proliferation and apoptosis are abnormal in FIV-infected cats in response to challenge with the intracellular pathogen, *Listeria monocytogenes*.

We have previously shown that the immune defect in FIV-infected cats in response to Lm infection occurs during the first four days after challenge and is characterized by a delayed cellular response in the draining lymph node and lack of control of bacterial growth [13]. However, FIV-infected cats are eventually able to clear the infection, presumably due to the T cell response, suggesting a defective innate immune response against Lm. We also have demonstrated that local delivery of IL-15 rescues the early response of FIV-infected cats to Lm, which correlates with increased NK cells in the lymph node. The biological activities of IL-15 include NK cell activation, proliferation, and increased cell survival [20].

*Listeria* infection is known to induce a multifactorial immune response that initially controls and eventually eliminates the bacterium. Early bacterial control does not seem to rely on T cells, since mice with severe combined immunodeficiency (SCID) control the initial infection as effectively as normal animals, yet ultimately fail to clear infection [21]. NK cells are thought to be an important component of the innate immune response to Lm through their production of IFN-γ induced by IL-12 and IL-18 [22]. Recently, athymic nude rats lacking T cells but with functional NK cells were shown capable of early control while rats depleted of NK cells were not [23]. Similarly, a previous study demonstrated that NK cell depletion led to a higher Lm burden in the draining lymph nodes after mice were subcutaneously inoculated [24]. Some studies have questioned the role played by NK cells during Lm infection. Teixeira and colleagues [25] showed that depletion of NK1^+^ cells in B6 mice led to a decreased Lm burden in the spleen, and soon after Takada and colleagues [26] confirmed this finding and suggested that γδ T cells play a role in the improved clearance of Lm infection. Despite the conflicting data demonstrating the importance of NK cells on the initial control of infection, it is a consensus that after Lm entry, NK cells are recruited from the blood to spleen and liver, and/or lymph nodes (depending on the route of inoculation), and an increase in NK cell activity during the first days of Lm infection is observed.

Challenge of SPF-control cats with Lm resulted in a 4-fold increase in draining lymph node cellularity characterized by significant increases in NK cells, CD4^+^ T cells and CD8^+^ T cells. Lymphocytes are recruited to the draining lymph node upon arrival of activated dendritic cells (DC) that produce chemokines, and this combined with a decrease in the efferent flow of lymph results in an increased cell number within the node [27]–[29]. DC also play a pivotal role in stimulating NK cell function and proliferation by means of cell-to-cell contact and cytokine production, including IL-15 and IL-12 [30]. The crosstalk between NK cells and DC following pathogen invasion is a key event in the induction of innate and adaptive immune responses [31]. One of the sites of DC-NK cell interaction is the secondary lymphoid tissue where activated NK cells produce cytokines, such as IFN-γ, that prime DC to produce IL-12 that in turn promotes further NK cell maturation as well as induction of CTL response [32], [33]. We evaluated the Langerhans cell (LC) population and found no quantitative difference between FIV-infected and SPF-control cats, suggesting that the defect in cell number is not due to reduced numbers of LC migrating to the draining LN. As we did not evaluate LC activation status or other DC populations, we cannot exclude the possibility that LC/DC dysfunction might play a role. It has been shown that plasmacytoid and myeloid DC are reduced in the LN of HIV-1 patients [3]. Another possible explanation is the altered expression of homing markers. Previous work showed NK cells from SIV-infected macaques upregulate the expression of the gut-homing marker α4β7 while CCR7 expression, a LN-trafficking marker, is downregulated [34]. Recently, a study showed NK cells from HIV-infected patients had reduced expression of the homing receptors CXCR1 and CX_3_CR1 [35], again suggesting that NK cell migration to LN may be compromised.

The substitution of thymidine with BrdU into the DNA of the dividing cells ensures restricted labeling of dividing cells. BrdU is not only retained in cells that have undergone division in the presence of BrdU but it is also passed to daughter cells after free BrdU has been cleared from the blood. However, there are also limitations to the use of BrdU, such as the uncertainty of BrdU distribution amongst tissues and the bioavailability in different species after administration. In order to confirm the data generated by in vivo BrdU labeling, we also utilized the nuclear protein Ki-67, a reliable marker of mitosis since it is expressed during mitosis and it has a short half-life, providing a snapshot of the dividing cells at a given time. As no ideal method of detection of proliferation exists, assessing the same data with more than one technique make it more reliable. In this study, we observed that for the majority of the analyses, both techniques showed very similar results; albeit a significant difference was occasionally observed with BrdU incorporation and not with Ki-67 staining, or vice-versa, the trends were consistent.

The proliferation rate of T cells and NK cells in the control and Lm-draining lymph nodes of FIV-infected cats was significantly greater compared to that of SPF-control animals. In the case of CD4^+^ and CD8^+^ T cells, this was counter-balanced by higher levels of apoptosis, but this was not true for NK cells, which showed a similar level of apoptosis regardless of FIV status. Under conditions of Lm challenge, the proliferative rate of T cells further increased in FIV-infected and uninfected cats indicating T cells were responsive to the immune challenge. Thus, overall while constitutive levels of proliferation and apoptosis of T cells were greater in FIV-infected cats, the T cell proliferative and apoptotic rates in response to the Lm challenge were similar to those of SPF-control cats. In contrast, the NK cell response was disparate between FIV-infected and control groups. NK cells from FIV-infected cats did not proliferate significantly in response to Lm challenge and did not down-regulate apoptosis as was observed in SPF-control cats. A higher constitutive turnover of NK and T cells has been shown in SIV-infected macaques [14], [36] and HIV-infected patients [37]. The rate of cell turnover has been correlated with plasma viremia [14], [37], [38], and is most likely a consequence of generalized immune activation [39], [40]. The lack of increased LN cellularity upon challenge of FIV-infected cats with Lm might suggests that cell turnover increased as proliferation increased. Although the present study was not designed to measure the turnover rate of cells, it did provide a snapshot of proliferation and apoptosis at a critical point during the innate immune response against Lm, revealing a disparate response between T cells and NK cells in FIV-infected and uninfected cats.

We showed that NK cells from FIV-infected cats failed to upregulate expression of perforin compared to SPF-control cats. NK cells are known for their ability to eliminate virus-infected or transformed cells by secreting pre-formed granules containing perforin and the serine protease granzymes, which combine to promote apoptosis of target cells [41]. Perforin is an essential component of cytotoxic cells and unlike granzymes, does not have any functional redundancy [42]. Furthermore, it has been shown that perforin knockout mice have an impaired ability to control Lm infection [43]. Previous reports have demonstrated that NK cells from chronic HIV-1 patients have low perforin expression and this is associated with NK cell anergy [44]. Based on our findings, we speculate that due to lower perforin production, NK cells from FIV-infected cats may not be capable of efficiently killing infected or transformed cells in vivo.

The observations that the NK cell response in FIV-infected cats was defective at the level of number, proliferation, apoptosis and function, combined with our previous observations that exogenous IL-15 rescued NK cell number and bacterial clearance, lead us to speculate that a qualitative or quantitative IL-15 deficiency may underlie the impaired innate immune response of FIV-infected cats to Lm challenge. This remains to be proven but if true, the spotlight would turn to the sources of IL-15: dendritic cells and macrophages. Given the general lack of response in the LN of FIV-infected cats, it seems plausible that there may be a more basic defect in the innate immune response, possibly at the dendritic cell level.

## Methods

### Ethics Statement

Animals were housed and cared for in accordance with Association for the Assessment of Laboratory Animal Care standards and Institutional Animal Care and Use Committee guidelines.

### Animals, Virus and Bacterial Inoculum, and BrdU Administration

Twenty-one specific-pathogen-free (SPF) cats were purchased from Liberty Labs (Liberty, Waverly, NY). Eleven neutered males and 2 females were infected with 5×10^5^ FIV cell-associated NCSU_1_ virus [45] by intravenous route at 20 weeks of age, and were considered chronically infected 12 months post-inoculation. Control cats included 3 neutered males and 5 females. A dose of 2.5×10^5^ cfu Lm was subcutaneously injected proximal to either the right metatarsal or metacarpal footpad of FIV-infected and control cats. A 20 mg/mL stock solution of 5-bromo-2′-deoxyuridine (BrdU) (Sigma, St. Louis, MO) was prepared by adding 1×PBS (Gibco, Life Technologies, Grand Island, NY). All cats received 30 mg/kg BrdU, intraperitoneally, on days 0, 1 and 2 post-Lm inoculation.

### Sample Collection, Processing, and Bacterial Quantification

Whole blood and either popliteal or cervical LN were collected 3 days post-Lm challenge. Peripheral blood mononuclear cells (PBMC) were isolated as previously described [46]. Blood for plasma isolation, complete blood counts, and leukocyte differentials was collected in EDTA tubes. Plasma was isolated by centrifugation and aliquots were stored at −80°C. Popliteal LN biopsies were performed on anesthetized cats while cervical LN were harvested at necropsy. Contralateral LN were harvested and used as controls. LN were weighted, bisected, and a portion was homogenized and cultured for Lm quantification as previously described [13], with the exception that LN homogenate was plated on BHI agar. The remaining portion of the LN was processed into single cell suspension as previously described [47] for flow cytometry or functional assay.

### Immunophenotyping

PBMC and LN cells (10^6^ cells) were labeled with the following monoclonal antibodies. Anti-CD3 (NZM1) [48] was used unconjugated, anti-CD4 (30A) [49] was conjugated to Pacific-Blue (Invitrogen, Life Technologies, Grand Island, NY), anti-CD25 (9F23) was conjugated to FITC using standard protocols, anti-CD8α (3.357) [49] was conjugated either to PerCP (ProZyme, Hayward, CA) or FITC, and anti-CD1a (Fel 5f4, Dr. Peter Moore, UC Davis) was conjugated to APC (ProZyme). Anti-CD4-PE (3–4F4; Southern Biotech, Birmingham, AL) and CD56-APC or PECy7 (HCD56; BioLegend, San Diego, CA) were purchased. Intracellular FOXP3 staining was performed using FIXP3-APC antibody as previously described [50]. Intranuclear BrdU staining was performed with BD Biosciences BrdU staining buffers and anti-BrdU-FITC antibody (3D4; San Diego, CA) according to manufacturer’s recommendations, with Ki-67-PE (B56, BD Biosciences) staining performed at the same time. AnnexinV (Invitrogen) was used according to manufacturer’s instructions. Staining and flow cytometric analysis were performed as described [51], with at least 500,000 gated events collected for each sample.

### Functional Assessment of NK Cells

The ability of ex vivo NK cells to produce perforin, granzyme A and IFN-γ was assessed using a FACS-based assay. Whole cell suspensions from LN were cultured overnight with and without 50 U/mL IL-2 in RPMI 1640 medium supplemented with 10% fetal bovine serum (Gibco), penicillin-streptomycin (10 IU/mL and 10 µg/mL respectively; Gibco), L-glutamine (4 mM; Invitrogen), sodium pyruvate (1 mM, Gibco), HEPES (15 mM, Gibco) and 2-mercaptoethanol (Gibco) and used as effector cells. Heterologous target cells were isolated from the spleen of a random source cat using anti-CD21 (1D6, Dr. Peter Moore) and anti-mouse Ig magnetic beads (Miltenyi Biotec Inc, Auburn, CA), and confirmed to be >95% B-cells. Cells were co-cultured at an effector:target ratio of 50∶1. Culture of effector cells without target was also assessed. Cells were cultured for 4h, with monensin (BioLegend) to a final concentration of 1×added after 1 h. Following culture, staining was completed as previously described [52] for NK cell surface receptors and intracellular production of perforin (DG9-PE, BioLegend), granzyme (CB9-Pacific Blue, BioLegend) and IFN-γ (E6D4A5-APC). Samples were analyzed using a BD LSRII flow cytometer, with a minimum of 500,000 gated events collected per sample.

### Viral Parameters

Quantitative real-time one-step reverse transcriptase (RT)-PCR assays were performed to detect viral RNA in plasma using NCSU_1_ FIV-gag specific primers and probe as previously described [50] (range of detection 10^1^ to 10^5^ copies/mL). FIV copies/mL plasma ranged between 471 and 5121 in FIV-infected cats (data not shown).

FIV proviral load was determined by real-time PCR using specific FIV-gag and CCR5 primers and probes as previously described [53], [54]. The limit of detection is ≤10 copies of FIV per 1 µg DNA. Proviral copies per 1×10^6^ cells ranged from 245 to 7345 in FIV-infected cats (data not shown).

### Statistical Analysis

Comparison between control and FIV-infected cats was performed by Mann-Whitney U test and Wilcoxon Signed-Rank test was used to compare Lm-challenged and control nodes between animals with the same FIV status. Means with standard errors (SEM) are reported. Significance was defined as *P*≤0.05 and analyses were performed using GraphPad Prism version 5.0 (GraphPad Software, La Jolla, CA).

## Supporting Information

Figure S1 **Relative percentages of LN cell populations after Lm challenge.** Chronically FIV-infected and SPF-control cats were challenged with 2.5×10^5^ cfu *Listeria monocytogenes* subcutaneously proximal to either the right metatarsal or metacarpal footpad. After 3 days, the local draining lymph node and the contralateral control node were removed, processed into a single cell suspension, assessed by trypan blue dye exclusion and the total number of cells per lymph node was determined. The percentages of lymphocyte subpopulations were determined by flow cytometric analysis. (A) Percent of NK cells, (B) Percent of NKT cells, (C) Percent of CD4^+^ T cells, (D) Percent of CD8^+^ T cells, and (E) Percent of Langerhans cells. Columns represent mean and standard error of the mean (SEM). FIV-infected (black columns) and, SPF-control animals (white columns). Statistical significance was determined between control and Lm challenged LN. Statistical analysis was performed using Wilcoxon Signed-Rank test. SPF-control cats n = 8, FIV-infected cats n = 13.
(TIF)

Figure S2 **Effect of FIV infection on PBMC subpopulations.** Chronically FIV-infected and SPF-control cats were challenged with 2.5×10^5^ cfu *Listeria monocytogenes* subcutaneously proximal to either the right metatarsal or metacarpal footpad. Whole blood was collected 3 days after challenge. The percentages of lymphocyte subpopulations were determined by flow cytometric analysis. (A) Percent of CD4^+^ T cells (B) Percent of CD8^+^ T cells, (C) Percent of NK cells. Columns represent mean ± SEM. FIV-infected (black columns) and SPF-control animals (white columns). Statistical significance was determined between control and Lm challenged LN. Statistical analysis was performed using Mann-Whitney U test. * indicates *P*<0.05. SPF-control cats n = 8, FIV-infected cats n = 13.
(TIF)

Figure S3 **Effect of FIV infection and Lm challenge on lymph node cell proliferation.** Cell proliferation was assessed by BrdU incorporation and expression of the nuclear antigen Ki-67. The percent of proliferating lymphocyte subpopulations was calculated based on the percent of gated lymphocytes that either incorporated BrdU or expressed Ki-67, determined by flow cytometric analysis and are shown within a given cell subpopulation. (A) Percent of proliferating NK cells, (B) Percent of proliferating CD4^+^ T cells, (C) Percent of proliferating CD8^+^ T cells, (D) Percent of total lymphocyte proliferating cells. Columns represent mean ± SEM. FIV-infected (black columns) and, SPF-control animals (white columns). Statistical significance was determined between control and challenged LN, and between FIV-infected and SPF-control cats. Statistical analysis was performed using Wilcoxon Signed-Rank test and Mann-Whitney U test. * indicates *P*<0.05, ** indicates *P*<0.01, *** indicates *P*<0.001. SPF-control cats n = 8, FIV-infected cats n = 13.
(TIF)

Figure S4 **Effect of FIV infection on peripheral blood mononuclear cell (PBMC) proliferation.** Whole blood was collected at the time of lymph node biopsy 3 days after the Lm challenge. Cell proliferation was assessed by BrdU incorporation and expression of the nuclear antigen Ki-67 and are shown within a given cell subpopulation. (A) Percent of proliferating NK cells, (B) Percent of proliferating CD4^+^ T cells, (C) Percent of proliferating CD8^+^ T cells, (D) Percent of proliferating total lymphocytes. Columns represent mean ± SEM. FIV-infected (black columns) and, SPF-control animals (white columns). Statistical significance was determined between FIV-infected and SPF-control cats. Statistical analysis was performed using Mann-Whitney U test. * indicates *P*<0.05. SPF-control cats n = 8, FIV-infected cats n = 13.
(TIF)

Figure S5 **Lymphocyte subpopulations from FIV-infected cats undergo apoptosis at a higher rate.** Apoptosis was assessed by AnnexinV staining followed by flow cytometric analysis. The percent of AnnexinV^+^ cells in lymphocyte subpopulations was determined by flow cytometric analysis. (A) Percent of AnnexinV^+^ of NK cells, (B) Percent of AnnexinV^+^ of CD4^+^ T cells, (C) Percent of AnnexinV^+^ of CD8^+^ T cells, (D) Percent of AnnexinV^+^ of total lymphocytes Columns represent mean ± SEM. FIV-infected (black columns) and, SPF-control animals (white columns). Statistical significance was determined FIV-infected and SPF-control cats. Statistical analysis was performed using Mann-Whitney U test. * indicates *P*<0.05, ** indicates *P*<0.01. SPF-control cats n = 5, FIV-infected cats n = 6.
(TIF)
