# Supplementary figures and images for: In Vivo Assessment of Natural Killer Cell Responses during Chronic Feline Immunodeficiency Virus Infection
Source: PLoS One. 2012 May 31;7(5):e37606. doi: 10.1371/journal.pone.0037606 (PMC3365115; doi:10.1371/journal.pone.0037606)

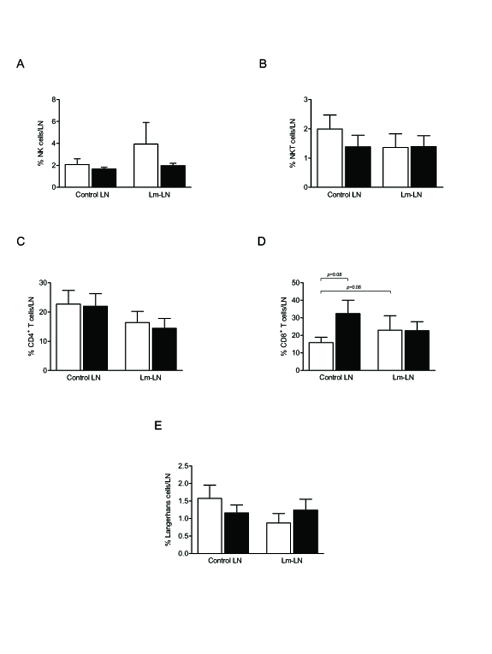

Supplement: Figure S1 — Relative percentages of LN cell populations after Lm challenge. Chronically FIV-infected and SPF-control cats were challenged with 2.5×10^5^ cfu Listeria monocytogenes subcutaneously proximal to either the right metatarsal or metacarpal footpad. After 3 days, the local draining lymph node and the contralateral control node were removed, processed into a single cell suspension, assessed by trypan blue dye exclusion and the total number of cells per lymph node was determined. The percentages of lymphocyte subpopulations were determined by flow cytometric analysis. (A) Percent of NK cells, (B) Percent of NKT cells, (C) Percent of CD4+ T cells, (D) Percent of CD8+ T cells, and (E) Percent of Langerhans cells. Columns represent mean and standard error of the mean (SEM). FIV-infected (black columns) and, SPF-control animals (white columns). Statistical significance was determined between control and Lm challenged LN. Statistical analysis was performed using Wilcoxon Signed-Rank test. SPF-control cats n = 8, FIV-infected cats n = 13. (TIF) [file pone.0037606.s001.tif]

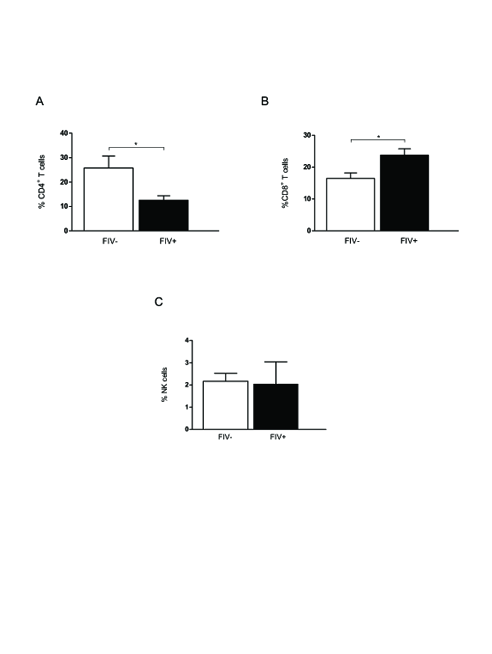

Supplement: Figure S2 — Effect of FIV infection on PBMC subpopulations. Chronically FIV-infected and SPF-control cats were challenged with 2.5×10^5^ cfu Listeria monocytogenes subcutaneously proximal to either the right metatarsal or metacarpal footpad. Whole blood was collected 3 days after challenge. The percentages of lymphocyte subpopulations were determined by flow cytometric analysis. (A) Percent of CD4+ T cells (B) Percent of CD8+ T cells, (C) Percent of NK cells. Columns represent mean ± SEM. FIV-infected (black columns) and SPF-control animals (white columns). Statistical significance was determined between control and Lm challenged LN. Statistical analysis was performed using Mann-Whitney U test. * indicates P<0.05. SPF-control cats n = 8, FIV-infected cats n = 13. (TIF) [file pone.0037606.s002.tif]

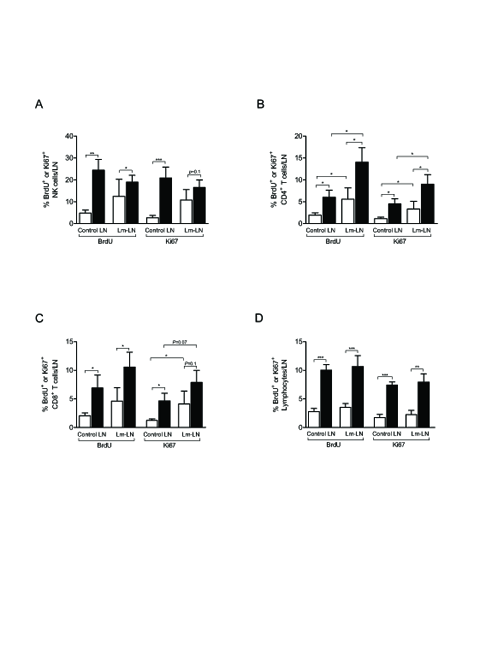

Supplement: Figure S3 — Effect of FIV infection and Lm challenge on lymph node cell proliferation. Cell proliferation was assessed by BrdU incorporation and expression of the nuclear antigen Ki-67. The percent of proliferating lymphocyte subpopulations was calculated based on the percent of gated lymphocytes that either incorporated BrdU or expressed Ki-67, determined by flow cytometric analysis and are shown within a given cell subpopulation. (A) Percent of proliferating NK cells, (B) Percent of proliferating CD4+ T cells, (C) Percent of proliferating CD8+ T cells, (D) Percent of total lymphocyte proliferating cells. Columns represent mean ± SEM. FIV-infected (black columns) and, SPF-control animals (white columns). Statistical significance was determined between control and challenged LN, and between FIV-infected and SPF-control cats. Statistical analysis was performed using Wilcoxon Signed-Rank test and Mann-Whitney U test. * indicates P<0.05, ** indicates P<0.01, *** indicates P<0.001. SPF-control cats n = 8, FIV-infected cats n = 13. (TIF) [file pone.0037606.s003.tif]

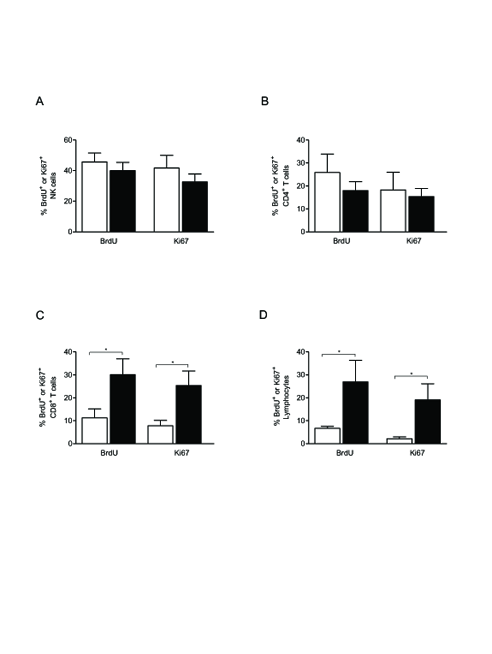

Supplement: Figure S4 — Effect of FIV infection on peripheral blood mononuclear cell (PBMC) proliferation. Whole blood was collected at the time of lymph node biopsy 3 days after the Lm challenge. Cell proliferation was assessed by BrdU incorporation and expression of the nuclear antigen Ki-67 and are shown within a given cell subpopulation. (A) Percent of proliferating NK cells, (B) Percent of proliferating CD4+ T cells, (C) Percent of proliferating CD8+ T cells, (D) Percent of proliferating total lymphocytes. Columns represent mean ± SEM. FIV-infected (black columns) and, SPF-control animals (white columns). Statistical significance was determined between FIV-infected and SPF-control cats. Statistical analysis was performed using Mann-Whitney U test. * indicates P<0.05. SPF-control cats n = 8, FIV-infected cats n = 13. (TIF) [file pone.0037606.s004.tif]

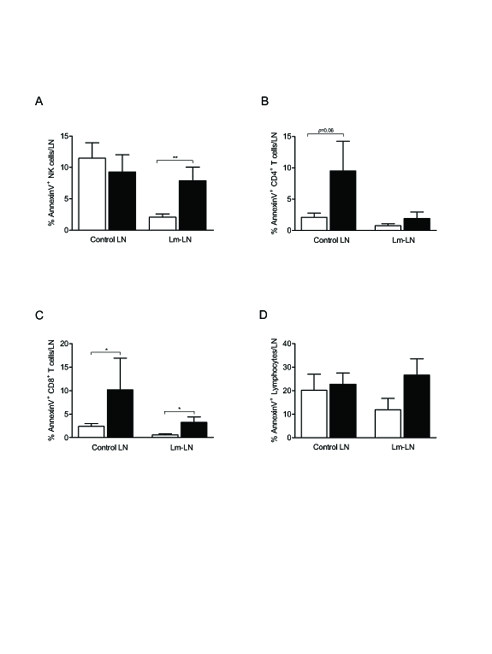

Supplement: Figure S5 — Lymphocyte subpopulations from FIV-infected cats undergo apoptosis at a higher rate. Apoptosis was assessed by AnnexinV staining followed by flow cytometric analysis. The percent of AnnexinV+ cells in lymphocyte subpopulations was determined by flow cytometric analysis. (A) Percent of AnnexinV+ of NK cells, (B) Percent of AnnexinV+ of CD4+ T cells, (C) Percent of AnnexinV+ of CD8+ T cells, (D) Percent of AnnexinV+ of total lymphocytes Columns represent mean ± SEM. FIV-infected (black columns) and, SPF-control animals (white columns). Statistical significance was determined FIV-infected and SPF-control cats. Statistical analysis was performed using Mann-Whitney U test. * indicates P<0.05, ** indicates P<0.01. SPF-control cats n = 5, FIV-infected cats n = 6. (TIF) [file pone.0037606.s005.tif]
